# Letter to Editor on "Impact of smart phone use on adolescence health in India"

**DOI:** 10.6026/973206300200036

**Published:** 2024-01-31

**Authors:** Bijurica Chakraborty, Rajib De

**Affiliations:** 1 Multi-disciplinary Research Unit (MRU), Nil Ratan Sircar Medical College & Hospital, 138 A.J.C. Bose Road, Kolkata-700014, India; 2 Department of Haematology, Nil Ratan Sircar Medical College & Hospital, 138 A.J.C. Bose Road, Kolkata-700014, India

**Keywords:** Letter to editor, views, smart phone, health

## Abstract

This letter to the editor with reference to Mahalakshmi *et al.* (2023) provides two additional views. In a tech-savvy world study in this field is of importance yet there is a huge gap. Such study should also consider screen time engagement of hospitalized patients given their predisposed physical condition in addition to student survey. Genetic analysis should also be included along with the questionnaire and counselling-based surveys. Thus, considering the known study pipeline and focusing on the two afore-mentioned aspects such research should be considered as a "High Priority" area.

## Background:

Recently I have come across a very interesting read entitled, "Impact of smart phone use on adolescence health in India," by Mahalakshmi *et al.* Bioinformation 19(11): 1090-1093 (2023). This article succinctly addresses the smartphone mediated multi-dimensional health impacts and influence of proper knowledge on its use on Indian adolescents, through a robust scoring approach. The sampling strategy followed a stratified random sampling, where n=60 voluntary student participants aged between 16-19 years, got enrolled. The scoring system was based on a statistical pre and post analysis. A 7-day gap following questionnaires on "Codex of Smartphone impact" (Helsinki Declaration of 1975 revised in 2013) was maintained between scores. The authors clearly stated the multiple health issues including disturbed sleep, negative impact on short term memory, distress, emotional and spiritual impairment through < 3 hours of smart phone use on an average each day. As former gadget is also a priority in terms of academics, knowledge gain, social connectivity or remaining up-to date, so the article concludes that "Codex on mobile phone use" followed by them was a statistically significant method (P<0.001*** level of significance) providing with satisfactory reduction in smartphone usage in their sampling cohort [1]. First, I would congratulate the authors for conducting this less explored yet very imperative a study in context of growing smart gadget usage in our country. In this letter specifically I would like to focus on two additional arms in this study for future consideration by researchers and funding agencies.

## Firstly, survey-based study for smart phone usage to be taken up as 'High Priority Research' for hospital admitted adolescents:

With increasing global interconnectivity an emerging tech-savvy population is at a surge [2]. Covid-19 scenario further accelerated the cell phone mediated negative effects [3]. In this context, as mentioned by Mahalakshmi *et al.*, India is second largest country in mobile usage and average family income sufficed an electronic gadget in participants. However, this study includes only students from educational institutes in India. Such surveys in line should also include adolescents admitted in hospitals for a prolonged period. A survey including usage of smart gadgets by inpatients (California) has been conducted before by Ludwin *et al.* (2015) and over two-third of the patients were found to use electronic gadgets during hospital stay [4]. As patients admitted in hospitals are already predisposed to certain physical ailments, so a thorough inspection for their screen time engagement is an urgent need of the hour. Patients who are admitted in hospital for prolonged period may consider android phones as a reasonable source of time pass. However, given their prior health condition, surveying additive negative impact imposed by excess screen time (if any) and optimizing usage of smart gadgets should be undertaken as "High Priority" research following similar protocol as described by Mahalakshmi *et al.* [1].

## Secondly, genetic analysis of smart phone users should be incorporated as a part of study

Several genetic associations with increased risk of mobile phone usage have been reported. Like people with high genetic risk of hypertension with longer use of mobile were more vulnerable and developed new episodes of tension as per a report [5]. Further an association between exposure to radio frequency radiation and genetic damage has been reported by another group [6]. Association between certain genetic variants with the risk of cell phone mediated thyroid cancer was also reported by Luo *et al.* [7]. As it has been rightly commented "for almost all human diseases, individual susceptibility is, to some degree, influenced by genetic variation" [8], so in addition to questionnaire and counseling-based survey, genetic analysis should also be included in such study. This will open a new avenue to decode many unanswered questions through genetic association and gene expression-based assays. Collection of samples (EDTA venous blood) will be convenient from both patients admitted in hospitals and institutional students. Thus, given an inevitable attachment of smart phones with our lives, where adolescents screen-time engagement are escalating, the issue of health impact and optimal use of gadget should be handled very sensitively yet with "High Priority" [1,9,10]. In such surveys I thus suggest to include adolescents from both the groups (a) Students (b) Hospitalized Patients. I also suggest incorporating "Genetic Analysis" as an important part of such study.
